# Supplementary material for: Plant invasion and naturalization are influenced by genome size, ecology and economic use globally
Source: Nat Commun. 2024 Feb 13;15:1330. doi: 10.1038/s41467-024-45667-4 (PMC10864296; doi:10.1038/s41467-024-45667-4)
Supplement: Supplementary file 1 — Supplementary Information [file 41467_2024_45667_MOESM1_ESM.pdf]

Supplementary Information

Plant invasion and naturalization are influenced by genome size, ecology and economic use globally

Kun Guo[1, 2], Petr Pyšek[3, 4], Mark van Kleunen[5, 6], Nicole L. Kinlock[5], Magdalena Lučanová[7, 8], Ilia J. Leitch[9], Simon Pierce[10], Wayne Dawson[11,12], Franz Essl[13], Holger Kreft[14, 15, 16], Bernd Lenzner[13], Jan Pergl[3], Patrick Weigelt[14, 15, 16], Wen-Yong Guo[1, 2, 17]*

[1]Zhejiang Tiantong Forest Ecosystem National Observation and Research Station, School of Ecological and Environmental Sciences, East China Normal University, 200241 Shanghai, P. R. China, email: kguo@geo.ecnu.edu.cn; wyguo@des.ecnu.edu.cn

[2]Research Center for Global Change and Complex Ecosystems, School of Ecological and Environmental Sciences, East China Normal University, 200241 Shanghai, P. R. China

[3]Czech Academy of Sciences, Institute of Botany, Department of Invasion Ecology, Průhonice, CZ-25243, Czech Republic, email: pysek@ibot.cas.cz; email: pergl@ibot.cas.cz

[4]Department of Ecology, Faculty of Science, Charles University, Viničná 7, Prague, CZ-12844, Czech Republic

[5]Ecology, Department of Biology, University of Konstanz, Universitätsstrasse 10, D-78457 Konstanz, Germany, email: mark.vankleunen@uni-konstanz.de; nicole.kinlock@uni-konstanz.de

[6]Zhejiang Provincial Key Laboratory of Plant Evolutionary Ecology and Conservation, Taizhou University, Taizhou 318000, P. R. China

[7]Czech Academy of Sciences, Institute of Botany, Department of Evolutionary Plant Biology, Průhonice, CZ-25243 Czech Republic, email: kubesovm@gmail.com

[8]Department of Botany, Faculty of Science, University of South Bohemia, Branišovská 1760, České Budějovice, CZ-370 05, Czech Republic

[9]Royal Botanic Gardens, Kew, Richmond, Surrey TW9 3AB, United Kingdom, email: i.leitch@kew.org

[10]Department of Agricultural and Environmental Sciences (DiSAA), University of Milan, Via G. Celoria 2, I-20133 Milan, Italy, email: simon.pierce@unimi.it

[11]Department of Biosciences, Durham University, Durham, United Kingdom

[12]Department of Evolution, Ecology and Behaviour, Institute of Infection, Veterinary and Ecological Sciences, University of Liverpool, Liverpool, UK, email: wayne.dawson@liverpool.ac.uk

[13]Division of BioInvasions, Global Change & Macroecology, Department of Botany and Biodiversity Research, University of Vienna, Vienna, Austria, email: franz.essl@univie.ac.at; bernd.lenzner@univie.ac.at

[14]Biodiversity, Macroecology & Biogeography, University of Goettingen, Göttingen, Germany, email: hkreft@uni-goettingen.de, pweigel@uni-goettingen.de

[15]Centre of Biodiversity and Sustainable Land Use (CBL), University of Goettingen, Göttingen, Germany

[16]Campus-Institute Data Science, Göttingen, Germany

[17]Shanghai Key Lab for Urban Ecological Processes and Eco-Restoration, School of Ecological and Environmental Sciences, East China Normal University, 200241 Shanghai, P. R. China

* Corresponding author: Wen-Yong Guo, wyguo@des.ecnu.edu.cn

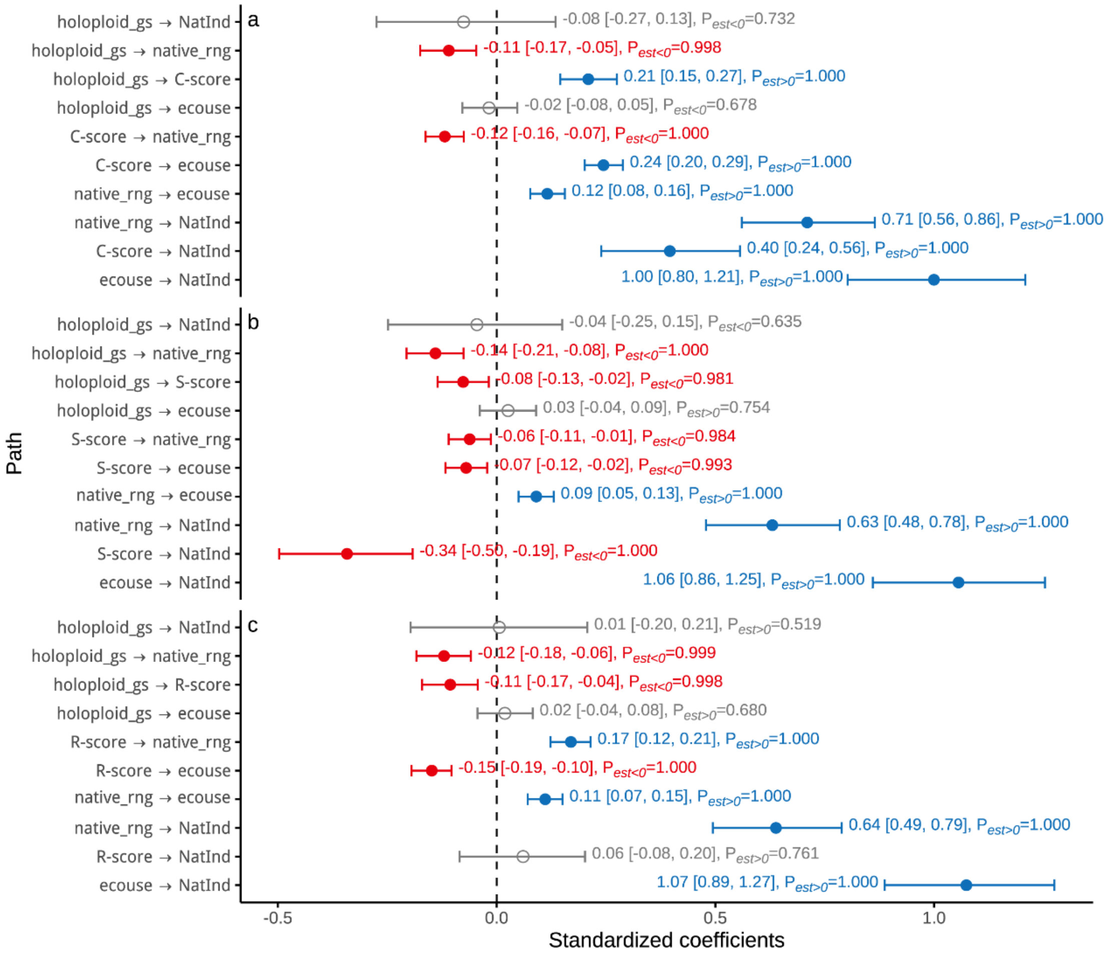

Supplementary Fig. 1 | Point plots showing the standardized coefficients (± 95% credible intervals) of each path in SEMs linking holoploid genome size to naturalization incidence (NatInd; whether the species is listed as naturalized in the GloNAF database; n = 1612). The filled red points, filled blue points and non-filled points, respectively, stand for positive, negative and non-significant coefficients. Abbreviations used in the plot: holoploid_gs, holoploid genome size; native_rng, native range size; ecouse, economic use.

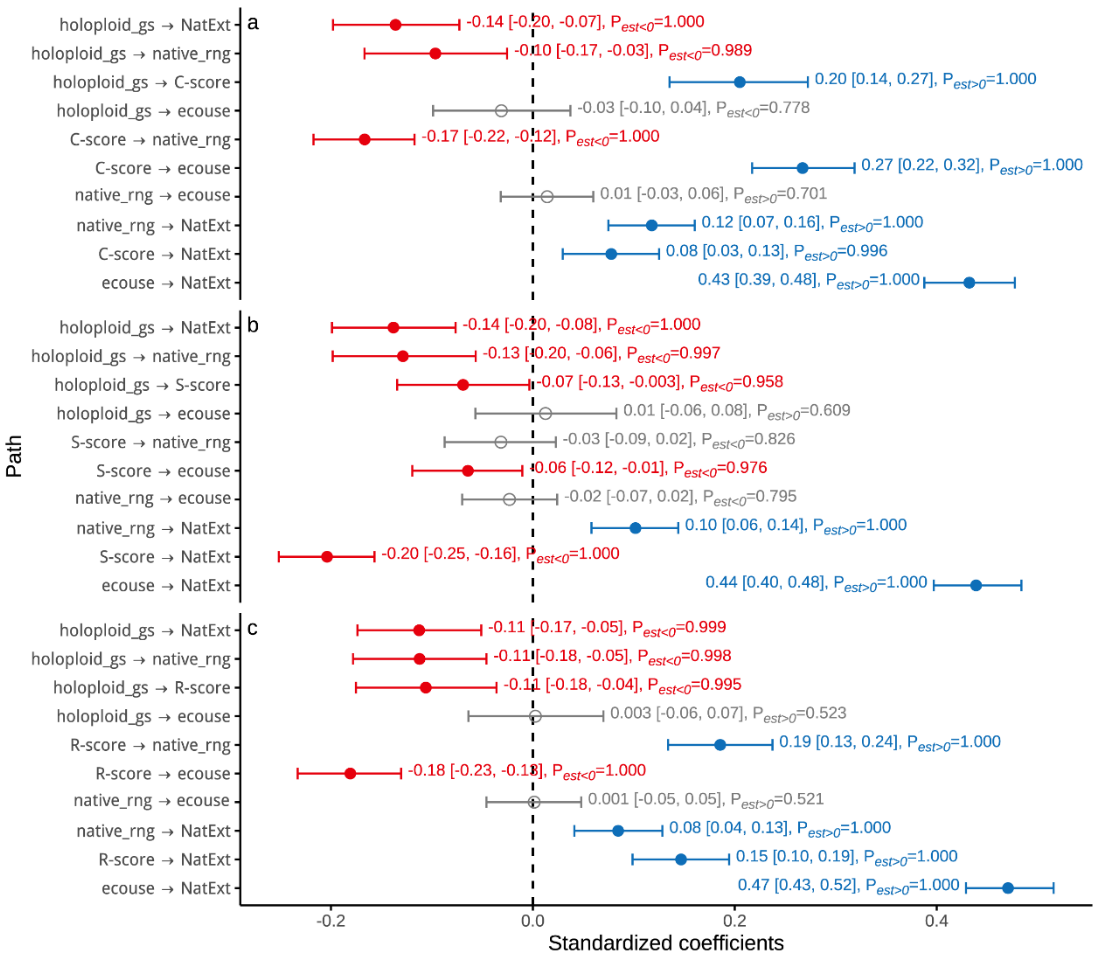

Supplementary Fig. 2 | Point plots showing the standardized coefficients (± 95% credible intervals) of each path in SEMs linking holoploid genome size to naturalization extent (NatExt; the number of regions a species has been recorded in as naturalized; n = 1193). The filled red points, filled blue points and non-filled points, respectively, stand for positive, negative and non-significant coefficients. Abbreviations used in the plot: holoploid_gs, holoploid genome size; native_rng, native range size; ecouse, economic use.

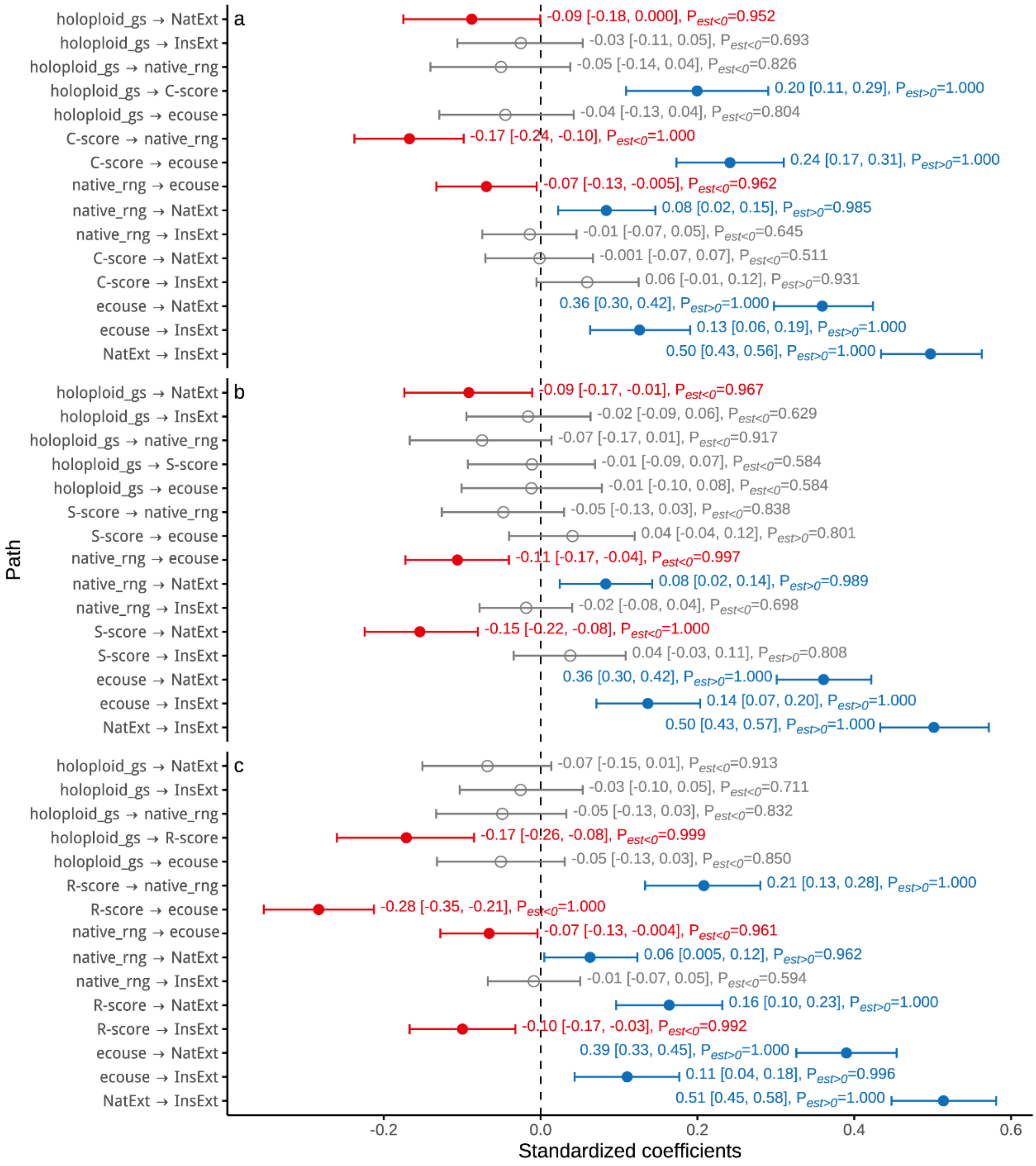

Supplementary Fig. 3 | Point plots showing the standardized coefficients (± 95% credible intervals) of each path in SEMs linking holoploid genome size to invasion extent (InvExt; the number of regions a species has been recorded in as invasive; n = 618). The filled red points, filled blue points and non-filled points, respectively, stand for positive, negative and non-significant coefficients. Abbreviations used in the plot: holoploid_gs, holoploid genome size; native_rng, native range size; ecouse, economic use; NatExt, naturalization extent.

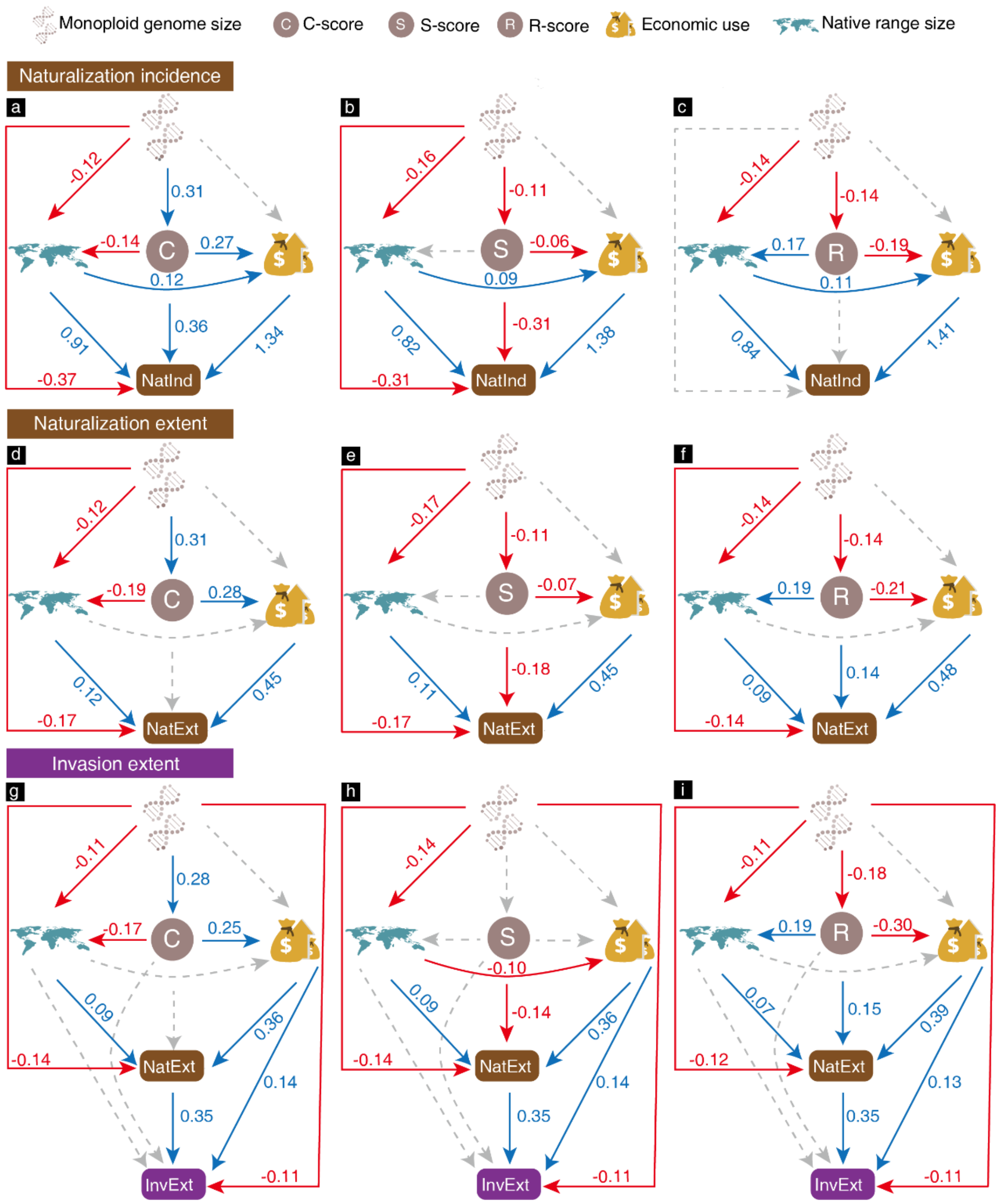

Supplementary Fig. 4 | Structural equation models linking plant monoploid genome size, CSR-strategy scores, native range size, and economic use to (a-c) naturalization incidence (NatInd, whether the species is listed as naturalized in the GloNAF database; n = 993), **(d-f) naturalization extent** (NatExt, the number of regions a species has been recorded in as naturalized; n = 792), and **(g-i) invasion extent** (InvExt, the number of regions a species has been recorded in as invasive; n = 450). The solid red line, solid blue line and dashed grey line, respectively, indicate negative, positive and nonsignificant (95% credible interval excludes or includes zero) relationships. Numbers beside the arrows are standardized coefficients, which are only shown for significant paths. See Fig. S5-7 for details of each path.

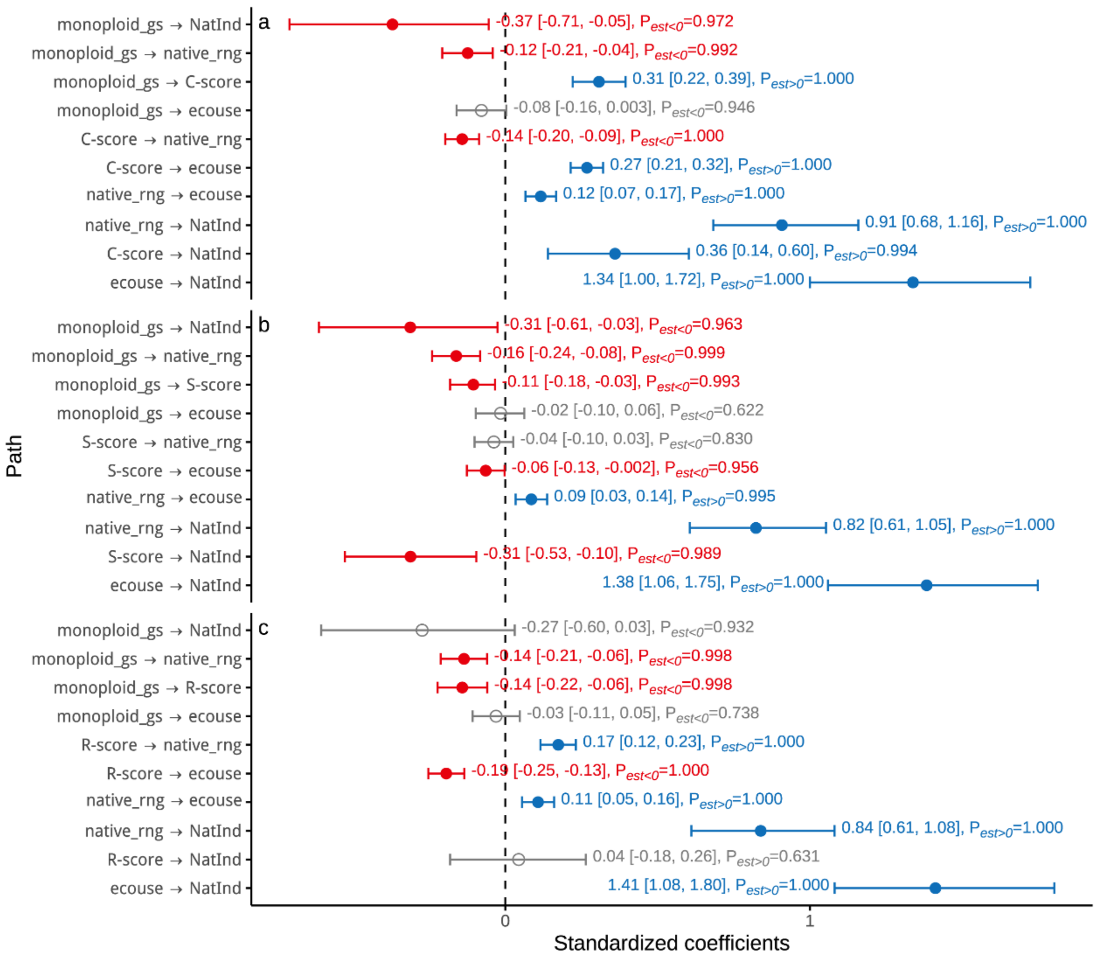

Supplementary Fig. 5 | Point plots showing the standardized coefficients (± 95% credible intervals) of each path in SEMs linking monoploid genome size to naturalization incidence (NatInd; whether the species is listed as naturalized in GloNAF database; n = 993). The filled red points, filled blue points and non-filled points, respectively, stand for positive, negative and non-significant coefficients. Abbreviations used in the plot: monoploid_gs, monoploid genome size; native_rng, native range size; ecouse, economic use.

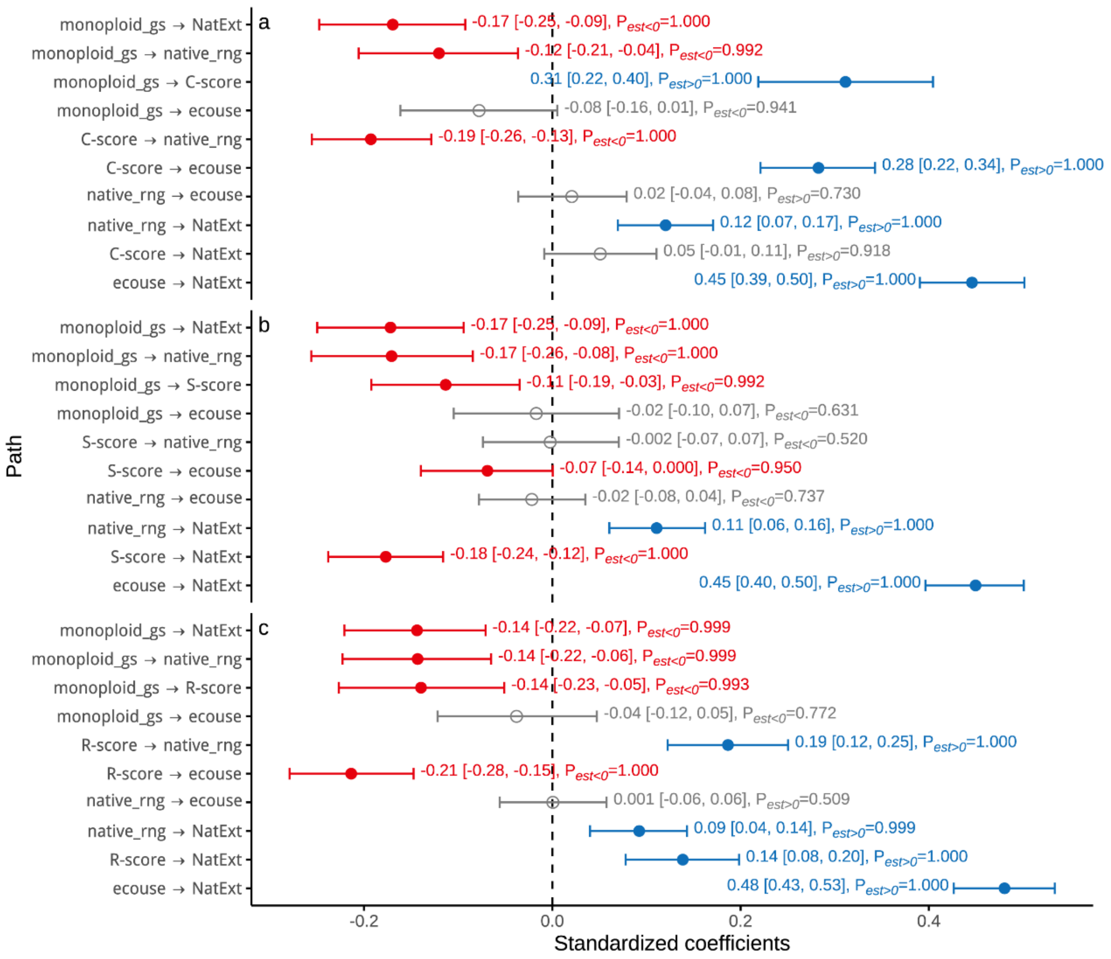

Supplementary Fig. 6 | Point plots showing the standardized coefficients (± 95% credible intervals) of each path in SEMs linking monoploid genome size to naturalization extent (NatExt; the number of regions a species has been recorded in as naturalized; n = 792). The filled red points, filled blue points and non-filled points, respectively, stand for positive, negative and non-significant coefficients. Abbreviations used in the plot: monoploid_gs, monoploid genome size; native_rng, native range size; ecouse, economic use.

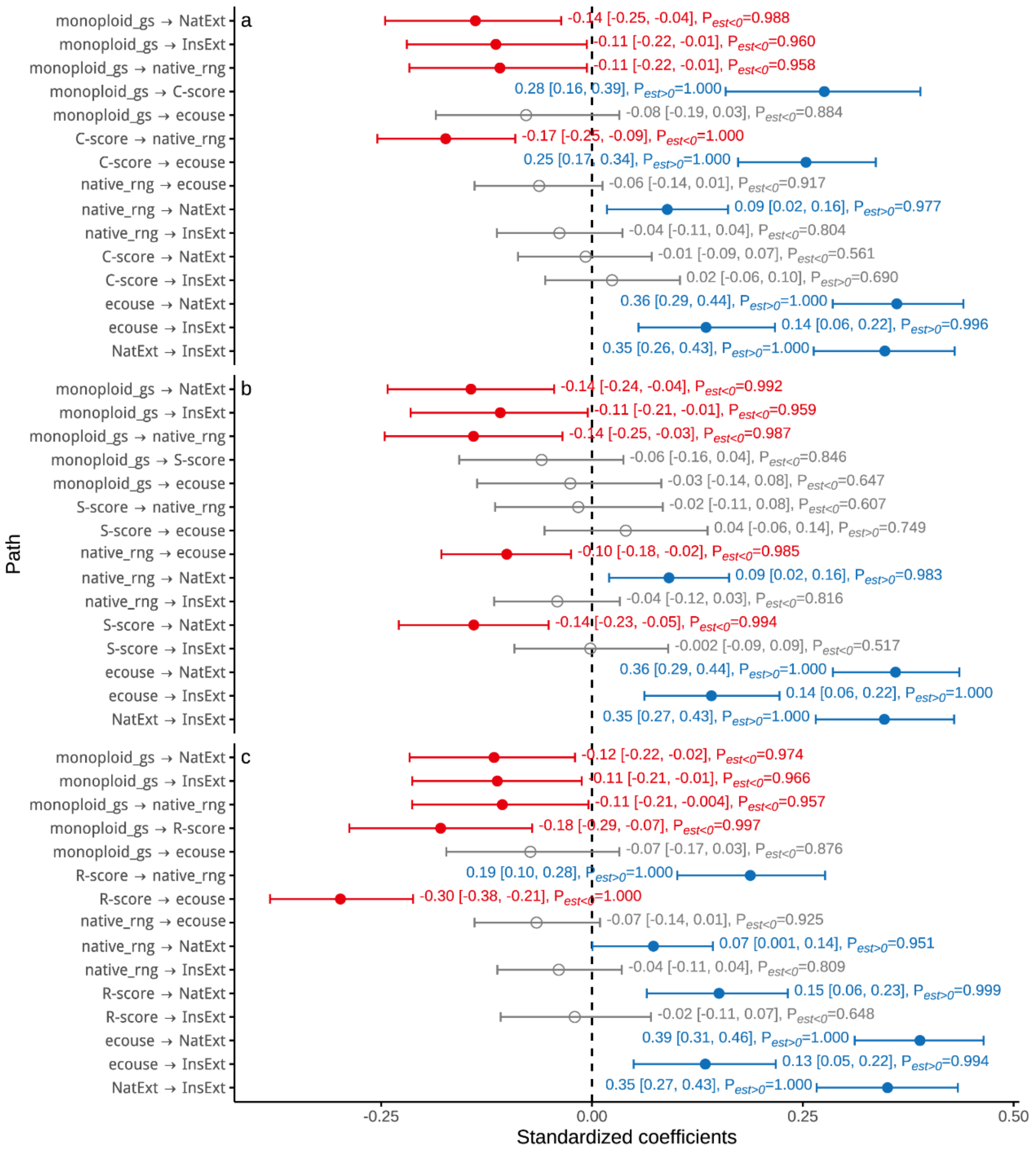

Supplementary Fig. 7 | Point plots showing the standardized coefficients (± 95% credible intervals) of each path in SEMs linking monoploid genome size to invasion extent (InvExt; the number of regions a species has been recorded in as naturalized; n = 450). The filled red points, filled blue points and non-filled points, respectively, stand for positive, negative and non-significant coefficients. Abbreviations used in the plot: monoploid_gs, monoploid genome size; native_rng, native range size; ecouse, economic use.

Supplementary Table 1 | Coefficients of direct and indirect paths from genome size to plant invasions (Fig. 3 and Supplementary Figure 4) identified from SEMs. We calculated the one-tailed probability that the estimated coefficients were > 0 ($P_{est}>0$, for positive estimates) or < 0 ($P_{est}<0$, for negative estimates). Non-significant coefficients of direct paths are marked with "NS", whereas only significant indirect paths are shown.

Figs	From	Via	To	Coefficients	
Fig. 3a	Holoploid genome size		Naturalization incidence	-0.080	NS
	Holoploid genome size	Native range size	Naturalization incidence	-0.078	
	Holoploid genome size	C-scores	Naturalization incidence	0.084	
	Holoploid genome size	C-scores → Native range size	Naturalization incidence	-0.018	
	Holoploid genome size	C-scores → Native range size → Economic uses	Naturalization incidence	-0.003	
	Holoploid genome size	C-scores → Economic uses	Naturalization incidence	0.050	
	Holoploid genome size	Native range size → Economic uses	Naturalization incidence	-0.013	
Fig. 3b	Holoploid genome size		Naturalization incidence	-0.040	NS
	Holoploid genome size	Native range size	Naturalization incidence	-0.088	
	Holoploid genome size	S-scores	Naturalization incidence	0.027	
	Holoploid genome size	S-scores → Native range size	Naturalization incidence	0.003	
	Holoploid genome size	S-scores → Native range size → Economic uses	Naturalization incidence	-0.0005	
	Holoploid genome size	S-scores → Economic uses	Naturalization incidence	0.006	
	Holoploid genome size	Native range size → Economic uses	Naturalization incidence	-0.013	
Fig. 3c	Holoploid genome size		Naturalization incidence	0.010	NS
	Holoploid genome size	Native range size	Naturalization incidence	-0.077	
	Holoploid genome size	R-scores	Naturalization incidence	-0.009	
	Holoploid genome size	R-scores → Native range size	Naturalization incidence	-0.012	
	Holoploid genome size	R-scores → Native range size → Economic uses	Naturalization incidence	-0.002	

	Holoploid genome size	R-scores → Economic uses	Naturalization incidence	0.018	
	Holoploid genome size	Native range size → Economic uses	Naturalization incidence	-0.014	
Fig. 3d	Holoploid genome size		Naturalization extent	-0.140	
	Holoploid genome size	Native range size	Naturalization extent	-0.012	
	Holoploid genome size	C-scores	Naturalization extent	0.016	
	Holoploid genome size	C-scores → Native range size	Naturalization extent	-0.004	
	Holoploid genome size	C-scores → Native range size → Economic uses	Naturalization extent	-0.0001	
	Holoploid genome size	C-scores → Economic uses	Naturalization extent	0.023	
	Holoploid genome size	Native range size → Economic uses	Naturalization extent	-0.001	
Fig.3e	Holoploid genome size		Naturalization extent	-0.140	
	Holoploid genome size	Native range size	Naturalization extent	-0.013	
	Holoploid genome size	S-scores	Naturalization extent	0.014	
	Holoploid genome size	S-scores → Economic uses	Naturalization extent	0.002	
Fig. 3f	Holoploid genome size		Naturalization extent	-0.110	
	Holoploid genome size	Native range size	Naturalization extent	-0.009	
	Holoploid genome size	R-scores	Naturalization extent	-0.017	
	Holoploid genome size	R-scores → Native range size	Naturalization extent	-0.002	
	Holoploid genome size	R-scores → Economic uses	Naturalization extent	-0.009	
Fig.3g	Holoploid genome size		Invasion extent	-0.090	NS
	Holoploid genome size	C-scores → Economic uses	Invasion extent	0.006	
	Holoploid genome size	C-scores → Economic uses → Naturalization extent	Invasion extent	0.009	
Fig.3h	Holoploid genome size		Invasion extent	-0.020	NS
	Holoploid genome size	Naturalization extent	Invasion extent	-0.045	
Fig.3i	Holoploid genome size		Invasion extent	-0.030	NS

	Holoploid genome size	R-scores	Invasion extent	0.017	
	Holoploid genome size	R-scores → Naturalization extent	Invasion extent	0.020	
	Holoploid genome size	R-scores → Native range size→ Naturalization extent	Invasion extent	0.000	
	Holoploid genome size	R-scores → Native range size → Economic uses	Invasion extent	0.000	
	Holoploid genome size	R-scores → Native range size → Economic uses → Naturalization extent	Invasion extent	0.000	
	Holoploid genome size	R-scores → Economic uses	Invasion extent	0.005	
	Holoploid genome size	R-scores → Economic uses → Naturalization extent	Invasion extent	0.009	
Fig.S4a	Monoploid genome size		Naturalization incidence	-0.370	
	Monoploid genome size	Native range size	Naturalization incidence	-0.109	
	Monoploid genome size	C-scores	Naturalization incidence	0.112	
	Monoploid genome size	C-scores → Native range size	Naturalization incidence	-0.039	
	Monoploid genome size	C-scores → Native range size → Economic uses	Naturalization incidence	-0.007	
	Monoploid genome size	C-scores → Economic uses	Naturalization incidence	0.112	
	Monoploid genome size	Native range size → Economic uses	Naturalization incidence	-0.019	
Fig.S4b	Monoploid genome size		Naturalization incidence	-0.310	
	Monoploid genome size	Native range size	Naturalization incidence	-0.131	
	Monoploid genome size	S-scores	Naturalization incidence	0.034	
	Monoploid genome size	S-scores → Economic uses	Naturalization incidence	0.009	
	Monoploid genome size	Native range size → Economic uses	Naturalization incidence	-0.020	
Fig.S4c	Monoploid genome size		Naturalization incidence	-0.270	NS
	Monoploid genome size	Native range size	Naturalization incidence	-0.118	
	Monoploid genome size	R-scores → Native range size	Naturalization incidence	-0.013	
	Monoploid genome size	R-scores → Native range size → Economic uses	Naturalization incidence	-0.004	
	Monoploid genome size	R-scores → Economic uses	Naturalization incidence	0.038	

	Monoploid genome size	Native range size → Economic uses	Naturalization incidence	-0.022
Fig.S4d	Monoploid genome size		Naturalization extent	-0.170
	Monoploid genome size	Native range size	Naturalization extent	-0.014
	Monoploid genome size	C-scores → Economic uses	Naturalization extent	0.038
Fig.S4e	Monoploid genome size		Naturalization extent	-0.170
	Monoploid genome size	Native range size	Naturalization extent	-0.019
	Monoploid genome size	S-scores	Naturalization extent	0.020
	Monoploid genome size	S-scores → Economic uses	Naturalization extent	0.003
Fig.S4f	Monoploid genome size		Naturalization extent	-0.140
	Monoploid genome size	Native range size	Naturalization extent	-0.013
	Monoploid genome size	R-scores	Naturalization extent	-0.020
	Monoploid genome size	R-scores → Native range size	Naturalization extent	-0.002
Fig.S4g	Monoploid genome size		Invasion extent	-0.110
	Monoploid genome size	Naturalization extent	Invasion extent	-0.049
	Monoploid genome size	Native range size	Invasion extent	0.004
	Monoploid genome size	Native range size → Naturalization extent	Invasion extent	-0.0004
	Monoploid genome size	C-scores → Native range size→ Naturalization extent	Invasion extent	-0.001
	Monoploid genome size	C-scores → Economic uses	Invasion extent	0.010
	Monoploid genome size	C-scores → Economic uses → Naturalization extent	Invasion extent	0.009
Fig.S4h	Monoploid genome size		Invasion extent	-0.110
	Monoploid genome size	Naturalization extent	Invasion extent	-0.049
	Monoploid genome size	S-scores → Economic uses → Naturalization extent	Invasion extent	0.002
	Monoploid genome size	Native range size → Economic uses	Invasion extent	0.002
	Monoploid genome size	Native range size → Economic uses → Naturalization extent	Invasion extent	0.002

Fig.4i	Monoploid genome size		Invasion extent	-0.110
	Monoploid genome size	Naturalization extent	Invasion extent	-0.064
	Monoploid genome size	Native range size → Naturalization extent	Invasion extent	-0.003
	Monoploid genome size	R-scores → Naturalization extent	Invasion extent	-0.009
	Monoploid genome size	R-scores → Native range size→ Naturalization extent	Invasion extent	-0.001
	Monoploid genome size	R-scores → Economic uses	Invasion extent	-0.006
	Monoploid genome size	R-scores → Economic uses → Naturalization extent	Invasion extent	0.007

Supplementary Table 2 | Description of the 10 main categories of economic use (derived from Diazgranados *et al.* 2020) employed in this study. Since the economic use categories 'gene sources' and 'poisons' do not necessarily require cultivation of the species, and because these categories were shown to contribute little to plant naturalization success (see Fig.2 in van Kleunen et al. 2020 for details), we excluded these two categories from the assessment of species economic use.

Economic use category	Abbreviation	Description
Animal food	AF	Forage and fodder for vertebrate animals only.
Environmental uses	EU	Intercrops and nurse crops, ornamentals, barrier hedges, shade plants, windbreaks, soil improvers, plants for revegetation and erosion control, wastewater purifiers, indicators of the presence of metals, pollution, or underground water.
Fuels	FU	Wood, charcoal, petroleum substitutes, fuel alcohols, etc. - have been separated from the 'MATERIALS' category below because of their importance.
Gene sources	GS	Wild relatives of major crops which may possess traits associated with biotic or abiotic resistance and may be valuable for breeding programs.
Human food	HF	Food, including beverages, for humans.
Invertebrate food	IF	Only plants eaten by invertebrates useful to humans, such as silkworms, lac insects, and edible grubs, are covered here.
Materials	MA	Woods, fibers, cork, cane, tannins, latex, resins, gums, waxes, oils, lipids, etc., and their derived products.
Medicines	ME	Both human and veterinary.
Poisons	PO	Plants which are poisonous to vertebrates and invertebrates, both accidentally and usefully, e.g., for hunting and fishing.
Social uses	SU	Plants used for social purposes, which are not definable as food or medicines, for instance, masticatories, smoking materials, narcotics, hallucinogens, psychoactive drugs, contraceptives, abortifacients, and plants with ritual or religious significance.

References:

Diazgranados, M., Allkin, B., Black, N., Cámara-Leret, R., Canteiro, C., Carretero, J., *et al.* (2020). World Checklist of Useful Plant Species. *Knowl. Netw. Biocomplexity*.

van Kleunen, M., Xu, X., Yang, Q., Maurel, N., Zhang, Z., Dawson, W., *et al.* (2020). Economic use of plants is key to their naturalization success. *Nat. Commun.*, 11, 1–12.
