# Supplementary material for: Plant invasion and naturalization are influenced by genome size, ecology and economic use globally
Source: Nat Commun. 2024 Feb 13;15:1330. doi: 10.1038/s41467-024-45667-4 (PMC10864296; doi:10.1038/s41467-024-45667-4)
Supplement: Supplementary file 2 — Reporting Summary [file 41467_2024_45667_MOESM2_ESM.pdf]

nature portfolio

Corresponding author(s): Wen-Yong Guo

Last updated by author(s): Jan 21, 2024

Reporting Summary

Nature Portfolio wishes to improve the reproducibility of the work that we publish. This form provides structure for consistency and transparency in reporting. For further information on Nature Portfolio policies, see our Editorial Policies and the Editorial Policy Checklist.

Statistics

For all statistical analyses, confirm that the following items are present in the figure legend, table legend, main text, or Methods section.

n/a	Confirmed	
☐	☒	The exact sample size (*n*) for each experimental group/condition, given as a discrete number and unit of measurement
☒	☐	A statement on whether measurements were taken from distinct samples or whether the same sample was measured repeatedly
☐	☒	The statistical test(s) used AND whether they are one- or two-sided<br>*Only common tests should be described solely by name; describe more complex techniques in the Methods section.*
☐	☒	A description of all covariates tested
☒	☐	A description of any assumptions or corrections, such as tests of normality and adjustment for multiple comparisons
☐	☒	A full description of the statistical parameters including central tendency (e.g. means) or other basic estimates (e.g. regression coefficient) AND variation (e.g. standard deviation) or associated estimates of uncertainty (e.g. confidence intervals)
☐	☒	For null hypothesis testing, the test statistic (e.g. *F*, *t*, *r*) with confidence intervals, effect sizes, degrees of freedom and *P* value noted<br>*Give P values as exact values whenever suitable.*
☐	☒	For Bayesian analysis, information on the choice of priors and Markov chain Monte Carlo settings
☒	☐	For hierarchical and complex designs, identification of the appropriate level for tests and full reporting of outcomes
☒	☐	Estimates of effect sizes (e.g. Cohen's *d*, Pearson's *r*), indicating how they were calculated

Our web collection on statistics for biologists contains articles on many of the points above.

Software and code

Policy information about availability of computer code

Data collection	All collected data was first processed in Microsoft Excel 365.
Data analysis	All data was analyzed using R version 4.1. We used R packages Taxonstand (version 2.1), V.PhyloMaker (version 0.1.0), brms (version 2.19.0), ggtree (version 3.6.2) and ape (version 5.7.1).

For manuscripts utilizing custom algorithms or software that are central to the research but not yet described in published literature, software must be made available to editors and reviewers. We strongly encourage code deposition in a community repository (e.g. GitHub). See the Nature Portfolio guidelines for submitting code & software for further information.

Data

Policy information about availability of data

All manuscripts must include a data availability statement. This statement should provide the following information, where applicable:

- Accession codes, unique identifiers, or web links for publicly available datasets
- A description of any restrictions on data availability
- For clinical datasets or third party data, please ensure that the statement adheres to our policy

The databases that we used are all publicly available: The Plant List (https://wfoplantlist.org/); the Plant DNA C-values database (https://cvalues.science.kew.org/); World Checklist of Useful Plant Species (WCUP, https://kew.iro.bl.uk/concern/datasets/7243d727-e28d-419d-a8f7-9ebef5b9e03e?); Kew Plants of the World (https://powo.science.kew.org); the Global Compositae Database (GCD, https://www.compositae.org); the USDA GRIN-Global (GRIN, https://npgsweb.ars-grin.gov/gringlobal/search); the IUCN Red list (IUCN, https://www.iucnredlist.org/); Global Naturalized Alien Flora [GloNAF]: https://doi.org/10.1002/ecy.2542); CABI

Invasive Species Compendium (https://www.cabi.org/isc); ISSG Global Invasive Species Database (https://www.iucngisd.org/gisd/); Smith and Brown phylogenetic tree: https://github.com/FePhyFoFum/big_seed_plant_trees. The data (.RData file) that support the findings of this study are available on Github (https://github.com/kun-ecology/WorldPlantInvasion) and are mirrored on Zenodo (https://doi.org/10.5281/zenodo.10113290).

Research involving human participants, their data, or biological material

Policy information about studies with human participants or human data. See also policy information about sex, gender (identity/presentation), and sexual orientation and race, ethnicity and racism.

Reporting on sex and gender	n.a.
Reporting on race, ethnicity, or other socially relevant groupings	n.a.
Population characteristics	n.a.
Recruitment	n.a.
Ethics oversight	n.a.

Note that full information on the approval of the study protocol must also be provided in the manuscript.

Field-specific reporting

Please select the one below that is the best fit for your research. If you are not sure, read the appropriate sections before making your selection.

☐ Life sciences ☐ Behavioural & social sciences ☒ Ecological, evolutionary & environmental sciences

For a reference copy of the document with all sections, see nature.com/documents/nr-reporting-summary-flat.pdf

Ecological, evolutionary & environmental sciences study design

All studies must disclose on these points even when the disclosure is negative.

Study description	We compiled a global dataset of 1,612 plant species with genome size, native range size, Grime's adaptive strategies, economic use, and information on their naturalization incidence and naturalization and invasion extent to explore how these multiple factors affect plant invasions worldwide. A hierarchical framework was developed and Bayesian structural equation models (SEMs) were used to test the intricate relationships conceptualized in the framework. SEMs were conducted on naturalization incidence, naturalization extent and invasion extent separately while accounting for phylogenetic relatedness.
Research sample	We used multiple datasets for data compilation, which include: the Plant List (https://wfoplantlist.org/); the Plant DNA C-values database (https://cvalues.science.kew.org/); World Checklist of Useful Plant Species (WCUP, https://kew.iro.bl.uk/concern/datasets/7243d727-e28d-419d-a8f7-9ebef5b9e03e?); Kew Plants of the World (https://powo.science.kew.org); the Global Compositae Database (GCD, https://www.compositae.org); the USDA GRIN-Global (GRIN, https://npgsweb.ars-grin.gov/gringlobal/search); the IUCN Red list (IUCN, https://www.iucnredlist.org); Global Naturalized Alien Flora [GloNAF]: https://doi.org/10.1002/ecy.2542); CABI Invasive Species Compendium (https://www.cabi.org/isc); ISSG Global Invasive Species Database (https://www.iucngisd.org/gisd/); Smith and Brown phylogenetic tree: https://github.com/FePhyFoFum/big_seed_plant_trees.
Sampling strategy	We merging data collected from different sources and only include species that have data for both economic uses and species characteristics for structural equation models.
Data collection	All authors were involved during the data collection and the data merging was performed by Kun Guo.
Timing and spatial scale	Data on genome size were extracted from the Plant DNA C-values database on August 22, 2021. Data on native range size were extracted from Kew Plants of the World, the Global Compositae Database, and the IUCN Red List at TDWG level 3 on November 29, 2021. Data on plant economic use were extracted from World Checklist of Useful Plant Species on June 20, 2022. The original data in the Global Naturalized Flora database comes from regional naturalized plant inventories collected from 1918 to 2018. Data on invasion extent were extracted from the CABI Invasive Species Compendium, the ISSG Global Invasive Species Database and the invasive plant species database at TDWG level 3 on January 1, 2022. There is no real rationale for the extraction date, we simply happened to have extracted the data on those dates.
Data exclusions	To be able to align the different databases, all taxonomic names were standardized according to The Plant List using R package Taxonstand. Taxa that did not have accepted names in The Plant List were excluded.
Reproducibility	All attempts to repeat the structural equation modeling and visualization were successfully when following associated protocols described in the Method section and using data and scripts provided in Data Availability and Code Availability sections.
Randomization	It is not relevant to our study since we don't need to use this method.
Blinding	It is not relevant to our study since we don't need to use this method.

Did the study involve field work? ☐ Yes ☒ No

Reporting for specific materials, systems and methods

We require information from authors about some types of materials, experimental systems and methods used in many studies. Here, indicate whether each material, system or method listed is relevant to your study. If you are not sure if a list item applies to your research, read the appropriate section before selecting a response.

Materials & experimental systems

n/a	Involved in the study
☒	☐ Antibodies
☒	☐ Eukaryotic cell lines
☒	☐ Palaeontology and archaeology
☒	☐ Animals and other organisms
☒	☐ Clinical data
☒	☐ Dual use research of concern
☒	☐ Plants

Methods

n/a	Involved in the study
☒	☐ ChIP-seq
☒	☐ Flow cytometry
☒	☐ MRI-based neuroimaging

Plants

Seed stocks	n.a.
Novel plant genotypes	n.a.
Authentication	n.a.
